# ABlooper: fast accurate antibody CDR loop structure prediction with accuracy estimation

**DOI:** 10.1093/bioinformatics/btac016

**Published:** 2022-01-31

**Authors:** Brennan Abanades, Guy Georges, Alexander Bujotzek, Charlotte M Deane

**Affiliations:** Department of Statistics, University of Oxford, Oxford, UK; Roche Pharma Research and Early Development, Large Molecule Research, Roche Innovation Center Munich, Penzberg, Germany; Roche Pharma Research and Early Development, Large Molecule Research, Roche Innovation Center Munich, Penzberg, Germany; Department of Statistics, University of Oxford, Oxford, UK

## Abstract

**Motivation:**

Antibodies are a key component of the immune system and have been extensively used as biotherapeutics. Accurate knowledge of their structure is central to understanding their antigen-binding function. The key area for antigen binding and the main area of structural variation in antibodies are concentrated in the six complementarity determining regions (CDRs), with the most important for binding and most variable being the CDR-H3 loop. The sequence and structural variability of CDR-H3 make it particularly challenging to model. Recently deep learning methods have offered a step change in our ability to predict protein structures.

**Results:**

In this work, we present ABlooper, an end-to-end equivariant deep learning-based CDR loop structure prediction tool. ABlooper rapidly predicts the structure of CDR loops with high accuracy and provides a confidence estimate for each of its predictions. On the models of the Rosetta Antibody Benchmark, ABlooper makes predictions with an average CDR-H3 RMSD of 2.49 Å, which drops to 2.05 Å when considering only its 75% most confident predictions.

**Availability and implementation:**

https://github.com/oxpig/ABlooper.

**Supplementary information:**

Supplementary data are available at *Bioinformatics* online.

## 1 Introduction

### 1.1 Antibody structure

Antibodies are a class of protein produced by B cells during an immune response. Their ability to bind with high affinity and specificity to almost any antigen makes them attractive for use as therapeutics (Carter and Lazar, 2018).

Knowledge of the structure of antibodies is becoming increasingly important in biotherapeutic development (Chiu *et al.*, 2019). However, experimental structure determination is time-consuming and expensive so it is not always practical or even possible to use routinely. Computational modelling tools have allowed researchers to bridge this gap by predicting large numbers of antibody structures to a high level of accuracy (Leem *et al.*, 2016; Ruffolo *et al.*, 2021). For example, models of antibody structures have recently been used for virtual screening (Schneider *et al.*, 2021) and to identify coronavirus-binding antibodies that bind the same epitope with very different sequences (Robinson *et al.*, 2021).

The overall structure of all antibodies is similar and therefore can be accurately predicted using current methods (e.g. Leem *et al.*, 2016). The area of antibodies that it is hardest to model is the sequence variable regions that provide the structural diversity necessary to bind a wide range of antigens. This diversity is largely focussed on six loops known as the complementarity determining regions (CDRs). The most diverse of these CDRs and therefore the hardest to model is the third CDR loop of the heavy chain (CDR-H3) (Teplyakov *et al.*, 2014).

### 1.2 Deep learning for protein structure prediction

At CASP14 (Kryshtafovych *et al.*, 2021), DeepMind showcased AlphaFold2 (Jumper *et al.*, 2021), a neural network capable of accurately predicting many protein structures. The method relies on the use of equivariant neural networks and an attention mechanism. More recently, RoseTTAFold, a novel neural network based on equivariance and attention was shown to obtain results comparable to those of AlphaFold2 (Baek *et al.*, 2021).

These methods both rely on the use of equivariant networks. For a network to be equivariant with respect to a group, it must be able to commute with the group action. For rotations, this means that rotating the input before feeding it into the network will have the same result as rotating the output. In the case of proteins, using a network equivariant to both translations and rotations in 3D space allows us to learn directly from atom coordinates. This is in contrast to previous methods like TrRosetta (Yang *et al.*, 2020) or the original version of AlphaFold (Senior *et al.*, 2020) that predicted invariant features, such as inter-residue distances and orientations which are then used to reconstruct the protein. A number of approaches for developing equivariant networks have been recently developed (e.g. Finzi *et al.*, 2021).

In this article, we explore the use of an equivariant approach to CDR structure prediction. We chose to use E(n)-Equivariant Graph Neural Networks (E(n)-EGNNs; Satorras *et al.*, 2021) as our equivariant approach due to their speed and simplicity.

### 1.3 Deep learning for antibody structure prediction

Deep learning-based approaches have also been shown to improve structure prediction in antibodies, e.g. DeepH3 (Ruffolo *et al.*, 2020), an antibody-specific version of TrRosetta. Recently, DeepAb (Ruffolo *et al.*, 2021), an improved version of DeepH3, was shown to outperform all currently available antibody structure prediction methods. DeepAb and DeepH3 are similar to TrRosetta and the original version of AlphaFold in that deep learning is used to obtain inter-residue geometries that are then fed into an energy minimization method to produce the final structure.

In this work, we present ABlooper, a fast and accurate tool for antibody CDR loop structure prediction. By leveraging E(n)-EGNNs, ABlooper directly predicts the structure of CDR loops. By simultaneously predicting multiple structures for each loop and comparing them amongst themselves, ABlooper is capable of estimating a confidence measure for each predicted loop.

## 2 Materials and Methods

### 2.1 Data

The data used to train, test and validate ABlooper were extracted from SAbDab (Dunbar *et al.*, 2014), a database of all antibody structures contained in the PDB (Berman *et al.*, 2000). Structures with a resolution better than 3.0 Å and no missing backbone atoms within any of the CDRs were selected. The CDRs were defined using the Chothia numbering scheme (Chothia *et al.*, 1989).

For easy comparison with different pipelines, we used the 49 antibodies from the Rosetta Antibody Benchmark as our test set. For validation, 100 structures were selected at random. It was ensured that there were no structures with the same CDR sequences in the training, testing and validation sets. Sequence redundancy was allowed within the training set to expose the network to the existence of antibodies with identical sequences but different structural conformations. This resulted in a total of 3438 training structures.

Additionally, we use a secondary test set composed of 114 antibodies (SAbDab Latest Structures) with a resolution of under 2.3 Å and a maximum CDR-H3 loop length of 20, which were added to SAbDab after the initial test, train and validation sets were extracted (November 8, 2020 to May 24, 2021). A list containing the PDB IDs of all the structures used in the train, test, and validation sets is given in the Supplementary Material.

ABodyBuilder was used to build models of all the structures. Structural models were generated using the singularity version of ABodyBuilder (Leem *et al.*, 2016) (fragment database from July 8, 2021) excluding all templates with a 99% or higher sequence identity. ABlooper CDR models for the test sets were obtained by remodelling the CDR loops on ABodyBuilder models.

### 2.2 Deep learning

**Fig. 1.**
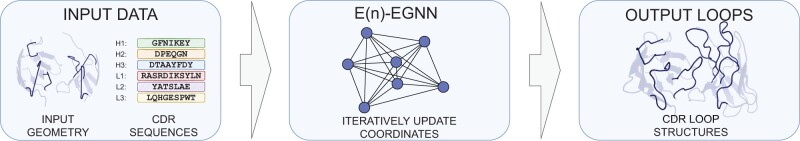
Flowchart showing how E(n)-EGNN is used to predict CDR loops in ABlooper. The input geometry for each CDR loop is generated by aligning its residues between their anchors, while the node features are extracted from the loop sequence. Atom coordinates are then iteratively updated using a four-layer E(n)-EGNN resulting in a predicted set of conformations for each CDR

ABlooper is composed of five E(n)-EGNNs, each one with four layers, all trained in parallel. The model is trained on the position of the $C_{\alpha }$-N-C-$C_{\beta }$ backbone atoms for all six CDR loops plus two anchor residues at either end. E(n)-EGNNs require a starting geometry, so a non-descriptive input geometry is generated by evenly spacing each CDR loop residue on a straight line between its anchor residues (Fig. 1). The model is given four different types of features per node resulting in a 41-dimensional vector. These include a one-hot encoded vector describing the amino acid type, the atom type and which loop the residue belongs to. Additionally, sinusoidal positional embeddings are given to each residue describing how close it is to the anchors. An outline of how E(n)-EGNNs are used within ABlooper is shown in Figure 1.

Two different losses were used during training. To quantify the structural similarity between the predicted and true structures, RMSD was used. To encourage the conservation of distances between neighbouring atoms in the backbone chain, an L1-loss between the true and predicted inter-atom distances was used. This was composed of five terms between the following pairs of atoms: $C_{\alpha }^{i}$-$C_{\alpha }^{i+1},\;C_{\alpha }^{i}$-$C_{\beta }^{i},\;C_{\alpha }^{i}$-$N^{i},\;C_{\alpha }^{i}$-$C^{i},\;C^{i}$-$N^{i+1}$.

Each of the five E(n)-EGNNs were trained to make predictions independently by minimizing the RMSD between their prediction and the crystal structure. The output from the five networks is then averaged to obtain a final prediction. To ensure that the final combined prediction of all E(n)-EGNNs was physically plausible, the L1-loss was used on the final averaged structure.

The model was trained in two phases. First, it was trained until convergence without the L1-loss term using the RAdam (Liu *et al.*, 2020) optimizer with a learning rate of $10^{-3}$ and a weight decay of $10^{-3}$. In the second stage, the L1-loss term was added with a weighting of 1.0. For this stage, the model was trained using the Adam (Kingma and Ba, 2014) optimizer with a learning rate of $10^{-4}$ and early stopping. More details on the implementation of ABlooper can be found in the Supplementary Material.

### 2.3 Loop relaxation

During training, ABlooper is encouraged to predict physically plausible CDR loops via the intra-residue atom distance loss term. However, ABlooper occasionally produces loops with incorrect backbone geometries. To enforce correct backbone geometries we relax the predicted loops using a restrained energy minimization procedure. As our energy function, we use the AMBER14 (Maier *et al.*, 2015) protein force field with an additional harmonic potential term keeping the positions of backbone atoms close to their original predicted positions. The spring constant of the harmonic potential is set to 10 kcal/mol^2^. Energy minimization is done using the Langevin Integrator in the OpenMM python package (Eastman *et al.*, 2017). This relaxation step typically results in a small loss in accuracy, but ensures that predicted loops are physically plausible.

### 2.4 Deepab and AlphaFold2

DeepAb structural models were generated using the open-source version of the code (available at https://github.com/RosettaCommons/DeepAb). As suggested in their paper (Ruffolo *et al.*, 2021), we generated five decoys per structure. This took around 10 min per antibody on an 8-core Intel i7-10700 CPU.

Antibody structures were generated using the open-source version of AlphaFold2 (available at https://github.com/deepmind/alphafold). We used the ‘full_dbs’ preset and allowed it to use templates from before May 14, 2020. As AlphaFold2 is intended to predict single chains (Jumper *et al.*, 2021), we predicted and aligned the heavy and light chain independently before comparing to other methods. On a 20-core Intel 6230 CPU this took around 3 h per antibody modelled.

## 3 Results

### 3.1 Using ABlooper to predict CDR loops on modelled antibody structures

We used ABlooper to predict the CDRs on ABodyBuilder models of the Rosetta Antibody Benchmark (RAB) and the SAbDab Latest Structures (SLS) sets. The RMSD between the $C_{\alpha }$-N-C-$C_{\beta }$ atoms in the backbone of the crystal structure and the predicted CDRs for both test sets is shown in Table 1.

**Table 1.** Performance comparison between AlphaFold2, ABodyBuilder, DeepAb and ABlooper for both test sets

Method	CDR-H1	CDR-H2	CDR-H3	CDR-L1	CDR-L2	CDR-L3
Rosetta Antibody Benchmark						
AlphaFold2^a^	0.84	0.99	2.87	0.53	0.49	0.95
ABodyBuilder	1.08	0.99	2.77	0.69	0.50	1.12
DeepAb	0.83	0.93	2.44	0.50	0.44	0.85
ABlooper	0.92	1.01	2.49	0.62	0.52	0.97
ABlooper unrelaxed	0.90	1.03	2.45	0.61	0.51	0.93
SAbDab latest structures						
ABodyBuilder	1.24	1.07	3.25	0.88	0.57	1.03
DeepAb^a^	1.00	0.82	2.49	0.59	0.45	0.90
ABlooper	1.14	0.97	2.72	0.74	0.55	1.04
ABlooper Unrelaxed	1.14	0.99	2.66	0.73	0.54	1.01

The mean RMSD to the crystal structure across each test set for the six CDRs is shown. RMSDs for each CDR are calculated after superimposing their corresponding chain to the crystal structure. RMSDs are given in Angstroms (Å).

a It is likely that AlphaFold2 used at least some of the structures in the benchmark set during training. Similarly, structures in the SAbDab Latest Structures set may have been used for training DeepAb.

ABlooper achieves lower mean RMSDs than AbodyBuilder for most CDRs (Table 1). By far, the largest improvement is for the CDR-H3 loop, where due to the large structural diversity, homology modelling performs worst (Leem *et al.*, 2016). ABlooper predicts loops of a similar accuracy to AlphaFold2 and DeepAb for all CDRs except CDR-H3, where ABlooper and DeepAb outperform AlphaFold2.

One potential source of error for ABlooper is the model frameworks generated by ABodyBuilder, so we examined its resilience to the small deviations seen in these models and found little to no correlation between framework error and CDR prediction error (see Supplementary Material).

### 3.2 Prediction diversity as a measure of prediction quality

**Fig. 2.**
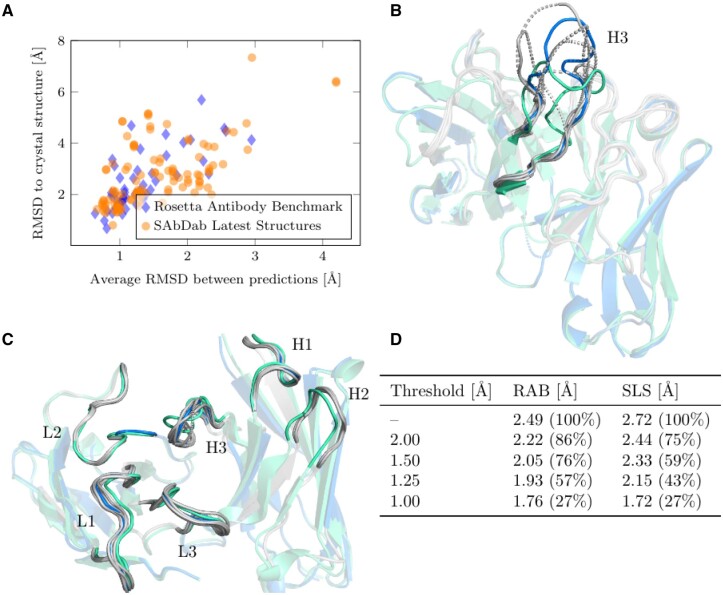
(**A**) CDR-H3 loop RMSD between final averaged prediction and crystal structure compared with average RMSD between the five ABlooper predictions for both the Rosetta Antibody Benchmark and the SAbDab Latest Structures set. (**B**) An example of a poorly predicted CDR-H3 loop. All five predictions are given in grey, with the final averaged prediction in blue and the crystal structure in green. The predictions from the five networks are very different, indicating an incorrect final prediction. (**C**) Example of correctly predicted CDR loops. All five predictions are similar, indicating a high confidence prediction. Colours are the same as in (B). (**D**) Effect of removing structures with a high CDR-H3 inter-prediction RMSD on the averaged RMSD for the set. The number of structures remaining after each quality cut-off is shown as a percentage. Data shown for the RAB and the SLS sets

ABlooper predicts five structures for each loop. We found that the average RMSD between predictions can be used as a measure of certainty of the final averaged prediction. If all five models agree on the same conformation, then it is more likely that it will be the correct conformation, if they do not, then the final prediction is likely to be less accurate (Fig. 2). This allows ABlooper to give a confidence score for each predicted loop. As shown in Figure 2D, this score can be used as a filter, removing structures which are expected to be incorrectly modelled by ABlooper. For example, by setting a 1.5 Å inter-prediction RMSD cut-off on structures from the Rosetta Antibody Benchmark, the average CDR-H3 RMSD for the set can be reduced from 2.49 to 2.05 Å while keeping around three quarters of the predictions. As expected, accuracy filtering has a tendency to remove longer CDR-H3 predictions but it is not exclusively correlated to length (see Supplementary Material).

## 4 Discussion

We present ABlooper, a fast and accurate tool for predicting the structures of the CDR loops in antibodies. It builds on recent advances in EGNNs to improve CDR loop structure prediction.

On an NVIDIA Tesla V100 GPU, the unrelaxed version of ABlooper can predict the CDR backbone atoms for 100 structures in under 5 s. Loop relaxation and side-chain prediction are the most computationally expensive parts of the pipeline taking around 10 s per structure. ABlooper outperforms ABodyBuilder (a state of the art homology method) and produces antibody models of similar accuracy to both AlphaFold2 and DeepAb, but on a far faster timescale.

By predicting each loop multiple times, ABlooper is capable of producing an accuracy estimate for each generated loop structure. It is not clear whether a high prediction diversity score is indicative of loops with multiple conformations or underrepresentation of the given loop sequence in SAbDab (Dunbar *et al.*, 2014). However, due to how ABlooper is trained (with the averaged prediction encouraged to be physically plausible), we would expect individual decoys from ABlooper to be unphysical for divergent predictions.

With the arrival of B-cell receptor repertoire sequencing, the number of publicly available paired antibody sequence data is rapidly increasing (Kovaltsuk *et al.*, 2018; Olsen *et al.*, 2022). Fast accurate tools such as ABlooper provide the opportunity for structural studies (such as Robinson *et al.*, 2021) at previously infeasible scales. The model used for ABlooper is available at: https://github.com/oxpig/ABlooper. 

## Funding

This work was supported by the Engineering and Physical Sciences Research Council (EPSRC) with grant number (EP/S024093/1).

*Conflict of Interest*: none declared.

## Supplementary Material

btac016_Supplementary_Data

## References

[btac016-B1] Baek M. et al (2021) Accurate prediction of protein structures and interactions using a three-track neural network. Science, 373, 871–876. 34282049 10.1126/science.abj8754 PMC7612213

[btac016-B2] Berman H.M. et al (2000) The Protein Data Bank. Nucleic Acids Res., 28, 235–242. 10592235 10.1093/nar/28.1.235 PMC102472

[btac016-B3] Carter P.J. , Lazar G.A. (2018) Next generation antibody drugs: pursuit of the ‘high-hanging fruit’. Nat. Rev. Drug Discov., 17, 197–223. 29192287 10.1038/nrd.2017.227

[btac016-B4] Chiu M.L. et al (2019) Antibody structure and function: the basis for engineering therapeutics. Antibodies, 8, 55. 31816964 10.3390/antib8040055 PMC6963682

[btac016-B5] Chothia C. et al (1989) Conformations of immunoglobulin hypervariable regions. Nature, 342, 877–883. 2687698 10.1038/342877a0

[btac016-B6] Dunbar J. et al (2014) SAbDab: the structural antibody database. Nucleic Acids Res., 42, D1140–D1146. 24214988 10.1093/nar/gkt1043 PMC3965125

[btac016-B7] Eastman P. et al (2017) OpenMM 7: rapid development of high performance algorithms for molecular dynamics. PLoS Comput. Biol., 13, e1005659. 28746339 10.1371/journal.pcbi.1005659 PMC5549999

[btac016-B8] Finzi M. et al (2021). A practical method for constructing equivariant multilayer perceptrons for arbitrary matrix groups. In: Proceedings of the 38th International Conference on Machine Learning, PMLR, Vol. 139, pp. 3318–3328.

[btac016-B9] Jumper J. et al (2021) Highly accurate protein structure prediction with alphafold. Nature, 596, 583–589. 34265844 10.1038/s41586-021-03819-2 PMC8371605

[btac016-B10] Kingma D.P. , Ba J. (2014). Adam: a method for stochastic optimization. *arXiv preprint arXiv:1412.6980.*

[btac016-B11] Kovaltsuk A. et al (2018) Observed antibody space: a resource for data mining next-generation sequencing of antibody repertoires. J. Immunol., 201, 2502–2509. 30217829 10.4049/jimmunol.1800708

[btac016-B12] Kryshtafovych A. et al (2021) Critical assessment of methods of protein structure prediction (CASP)—round XIV. Proteins, 89, 1607–1617. 34533838 10.1002/prot.26237 PMC8726744

[btac016-B13] Leem J. et al (2016). ABodyBuilder: automated antibody structure prediction with data–driven accuracy estimation. MAbs, 8, 1259–1268. 27392298 10.1080/19420862.2016.1205773 PMC5058620

[btac016-B14] Liu L. et al (2020). On the variance of the adaptive learning rate and beyond. In: Proceedings of the Eighth International Conference on Learning Representations (ICLR 2020).

[btac016-B15] Maier J.A. et al (2015) ff14SB: improving the accuracy of protein side chain and backbone parameters from ff99SB. J. Chem. Theory Comput., 11, 3696–3713. 26574453 10.1021/acs.jctc.5b00255 PMC4821407

[btac016-B16] Olsen T.H. et al (2022) Observed antibody space: a diverse database of cleaned, annotated, and translated unpaired and paired antibody sequences. Protein Sci., 31, 141–146. 34655133 10.1002/pro.4205 PMC8740823

[btac016-B17] Robinson,S.A. et al (2021) Epitope profiling using computational structural modelling demonstrated on coronavirus-binding antibodies. *PLoS Computational Biology*, 17, e1009675 34898603 10.1371/journal.pcbi.1009675 PMC8700021

[btac016-B18] Ruffolo J.A. et al (2020) Geometric potentials from deep learning improve prediction of CDR H3 loop structures. Bioinformatics, 36, i268–i275. 32657412 10.1093/bioinformatics/btaa457 PMC7355305

[btac016-B19] Ruffolo J.A. et al (2021) Antibody structure prediction using interpretable deep learning. Patterns, 100406. 35199061 10.1016/j.patter.2021.100406 PMC8848015

[btac016-B20] Satorras V.G. et al (2021). E (n) equivariant graph neural networks. *arXiv preprint arXiv:2102.09844.*

[btac016-B21] Schneider C. , et al. (2021). DLAB: deep learning methods for structure-based virtual screening of antibodies. Bioinformatics, 38(2), 377–383. 10.1093/bioinformatics/btab660 PMC8723137 34546288

[btac016-B22] Senior A.W. et al (2020) Improved protein structure prediction using potentials from deep learning. Nature, 577, 706–710. 31942072 10.1038/s41586-019-1923-7

[btac016-B23] Teplyakov A. et al (2014) Antibody modeling assessment II. structures and models. Proteins, 82, 1563–1582. 24633955 10.1002/prot.24554

[btac016-B24] Yang J. et al (2020) Improved protein structure prediction using predicted interresidue orientations. Proc. Natl. Acad. Sci. USA, 117, 1496–1503. 31896580 10.1073/pnas.1914677117 PMC6983395

