# Supplementary material for: ABlooper: fast accurate antibody CDR loop structure prediction with accuracy estimation
Source: Bioinformatics. 2022 Jan 31;38(7):1877–80. doi: 10.1093/bioinformatics/btac016 (PMC8963302; doi:10.1093/bioinformatics/btac016)
Supplement: btac016_Supplementary_Data [file btac016_supplementary_data.zip › btac016-suppl_data/ABlooper_SI.pdf]

Supplementary Information

A Network implementation

ABlooper was implemented in Pytorch [2]. The model is composed of five E(n)-EGNNs [5] all simultaneously predicting the structure of each loop. The implementation of E(n)-EGNN is based on the open source version of Phil Wang [6]. The main difference between our implementation of E(n)-EGNN and the original is the normalisation of relative coordinates before each coordinate update. This was found to help stability during training. Additionally, edge features were not used in our model. With these changes, the equations describing the E(n)-EGNN algorithm become:

$$\begin{aligned}
\mathbf{r_{ij}} &= \mathbf{x_i^l} - \mathbf{x_j^l} \\
\mathbf{m_{ij}} &= \phi_e(\mathbf{h_i^l}, \mathbf{h_j^l}, \|\mathbf{r_{ij}}\|^2) \\
\mathbf{x_i^{l+1}} &= \mathbf{x_i^l} + C \sum_{j \neq i} \mathbf{\hat{r}_{ij}}\, \phi_x(\mathbf{m_{ij}}) \\
\mathbf{m_i} &= \sum_j \mathbf{m_{ij}} \\
\mathbf{h_i^{l+1}} &= \mathbf{h_i^l} + \phi_h(\mathbf{h_i^l}, \mathbf{m_i})
\end{aligned}$$

Where $\mathbf{x_i^l}$ are the coordinates of node i after layer l and $\mathbf{h_i^l}$ is the 41-dimensional feature vector of node i. In this notation, ϕ is a Multi Layer Perceptron (MLP), C represents a learnable parameter at each layer and $\mathbf{\hat{r}_{ij}}$ is the normalised distance between the points i and j.

The edge MLP (ϕ_e), is composed of Linear-SiLU-Linear-SiLU layers, yielding 32 features for each pair of nodes. The node and coordinate update MLPs (ϕ_x and ϕ_h) are both composed of Linear-SiLU-Linear layers.

B Prediction dependence on framework quality

Here we explore whether the quality of the frameworks generated by ABodyBuilder affect the quality of ABlooper's CDR-H3 loop predictions. SI Figure 1 shows that, ABlooper is capable of consistently predicting accurate CDR-H3 loop structures independent of small errors in ABodyBuilder framework predictions.

C Prediction diversity against loop length

Longer CDR loops have been shown to be harder to model [3, 4]. As shown in SI Figure 2, longer loops tend to have higher prediction diversity. This

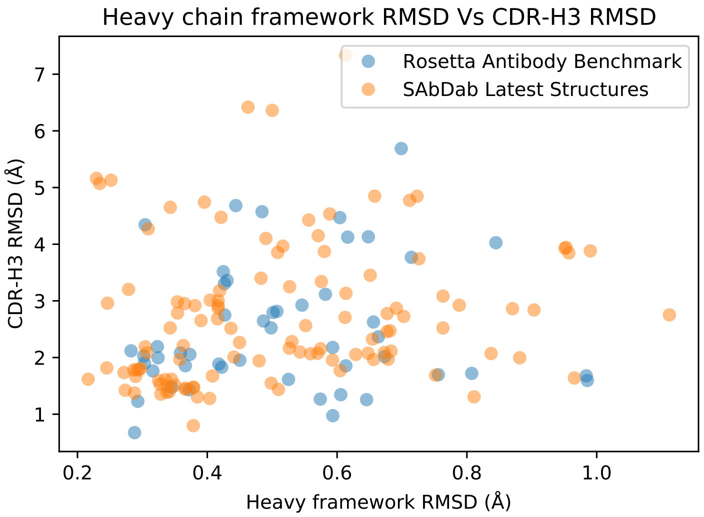

SI Figure 1: Heavy chain framework RMSD of the ABodyBuilder model against CDR-H3 RMSD of ABlooper Predictions. Framework RMSDs were calculated after superimposing the predicted ABodyBuilder framework onto the crystal structure.

means that accuracy filtering will tend to select shorter loops. However, we find that filtering by prediction diversity is effective even amongst loops of the same length. For example, if we select the 75% best scoring CDR-H3 predictions for each loop length separately, the average RMSD over both test sets drops from 2.65Å to 2.45Å.

D Prediction diversity as a measure of prediction quality for all CDR loops

As shown in Figure 1 of the manuscript, the accuracy of a CDR-H3 loop predicted by ABlooper can be estimated by the diversity of predicted conformations. In SI Figure 3, we show this relation for each of the CDR loops. In contrast to CDR-H3, the other CDR loops have a limited number of canonical forms [1] and because of this, the distributions for other CDRs follow a less clear trend than for CDR-H3.

Based on these plots, we explored a few cases in more detail. For example, in the case of the antibody with PDB code 3mlr, the CDR-L3 loops is predicted incorrectly (RMSD $\approx$ 8.6Å) with what could be considered an acceptable level of confidence. When looking at the predicted structure, it was found that this is due to ABodyBuilder incorrectly modelling the framework region. This shows the reliance of ABlooper on obtaining accurate templates for the framework.

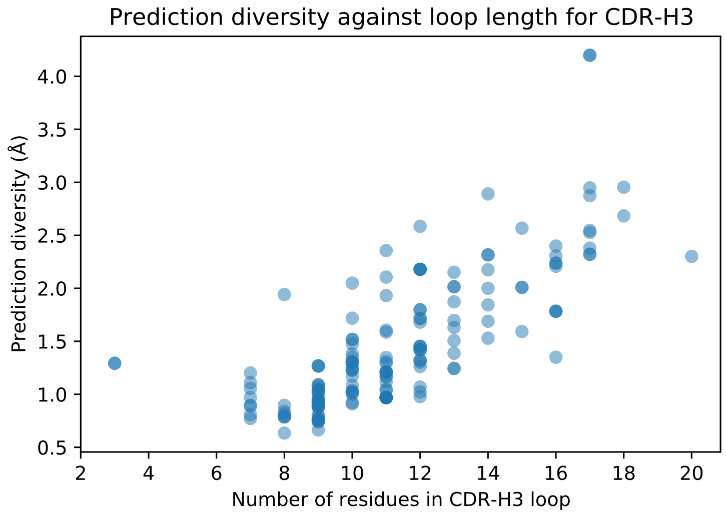

SI Figure 2: Prediction diversity RMSD against the number of amino acids in CDR-H3 loop for all of the structures in the RAB and the SLS datasets.

A second case is the model of the antibody with PDB code 3lmj, for which ABlooper obtains an RMSD of around 6.3Å for the CDR-H2 loop. In this example, the model incorrectly predicts an interaction between the CDR-H3 and CDR-H2 loops, displacing the CDR-H2. In this case the predicted accuracy is low.

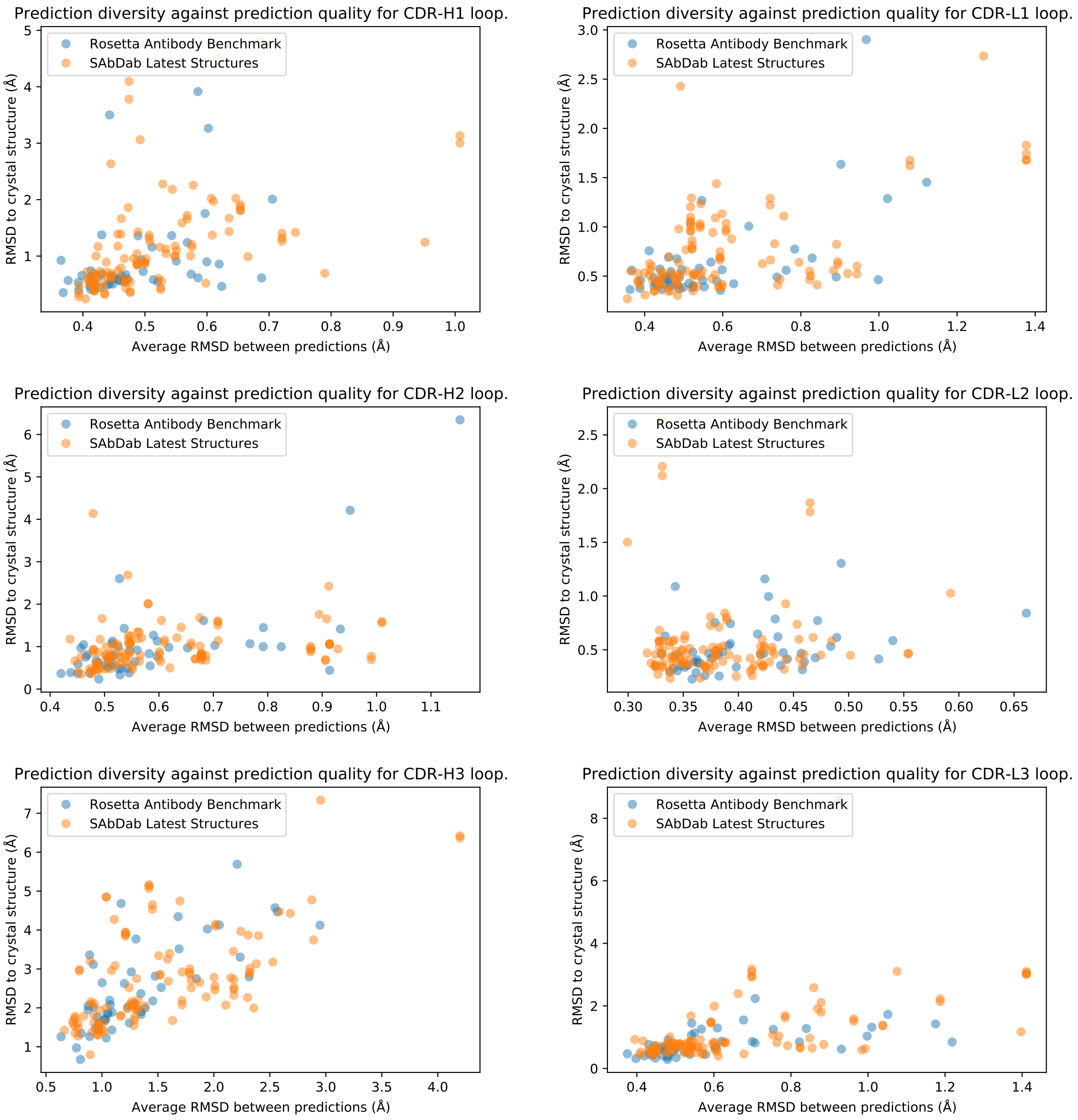

SI Figure 3: Prediction diversity against prediction quality for each of the six CDR loops.

References

[1] Cyrus Chothia, Arthur M Lesk, Anna Tramontano, Michael Levitt, Sandra J Smith-Gill, Gillian Air, Steven Sheriff, Eduardo A Padlan, David Davies,

William R Tulip, et al. Conformations of immunoglobulin hypervariable regions. *Nature*, 342(6252):877–883, 1989.

[2] Adam Paszke, Sam Gross, Francisco Massa, Adam Lerer, James Bradbury, Gregory Chanan, Trevor Killeen, Zeming Lin, Natalia Gimelshein, Luca Antiga, Alban Desmaison, Andreas Kopf, Edward Yang, Zachary DeVito, Martin Raison, Alykhan Tejani, Sasank Chilamkurthy, Benoit Steiner, Lu Fang, Junjie Bai, and Soumith Chintala. Pytorch: An imperative style, high-performance deep learning library. In H. Wallach, H. Larochelle, A. Beygelzimer, F. d'Alché-Buc, E. Fox, and R. Garnett, editors, *Advances in Neural Information Processing Systems 32*, pp. 8024–8035. Curran Associates, Inc., 2019.

[3] Jeffrey A Ruffolo, Carlos Guerra, Sai Pooja Mahajan, Jeremias Sulam, and Jeffrey J Gray. Geometric potentials from deep learning improve prediction of CDR H3 loop structures. *Bioinformatics*, 36(Supplement_1):i268–i275, 2020.

[4] Jeffrey A Ruffolo, Jeremias Sulam, and Jeffrey J Gray. Antibody structure prediction using interpretable deep learning. *bioRxiv*, 2021. 2021.05.27.445982.

[5] Victor Garcia Satorras, Emiel Hoogeboom, and Max Welling. E (n) equivariant graph neural networks. *arXiv preprint arXiv:2102.09844*, 2021.

[6] Phil Wang and Eric Alcaide. EGNN - Pytorch. `https://github.com/lucidrains/egnn-pytorch`, 2021.
